# Causal relationships between immunophenotypes, plasma metabolites, and temporomandibular disorders based on Mendelian randomization

**DOI:** 10.1038/s41598-024-73330-x

**Published:** 2024-09-27

**Authors:** Danqi Qiu, Shuntao Sun

**Affiliations:** Department of Stomatology, Hangzhou Children’s Hospital, Hangzhou, Zhejiang China

**Keywords:** Temporomandibular disorders, Mendelian randomization analysis, Immunophenotypes, Plasma metabolites, Mediation analysis, Immunology, Pathogenesis, Risk factors, Metabolism

## Abstract

While numerous studies have underscored the implication of immune cells and metabolites in temporomandibular disorders (TMD), conclusive evidence for causality remains elusive. Consequently, our aim is to explore the causal connections between immunophenotypes and plasma metabolites in relation to TMD employing a bidirectional Mendelian randomization (MR) approach. Summary statistics data on 731 immunophenotypes (n = 3757) and 1091 plasma metabolites (n = 8299) were obtained from comprehensive genome-wide association studies (GWAS), while TMD data (5668 cases and 205,355 controls) were acquired from the FinnGen Consortium. Bidirectional MR analyses and a two-step MR approach assessed causal relationships and potential intermediaries. Various corrections and sensitivity analyses were utilized to assess the robustness of the findings. Two immunophenotypes and seven metabolites were significantly associated with TMD risk. Specifically, Alpha-hydroxyisovalerate mediated the link between CD33 on CD33dim HLA DR + CD11b + and TMD (β = 0.034, *P* = 5.95 × 10^–5^), while CD8 on NKT cells mediated the causal relationship between 5-acetylamino-6-formylamino-3-methyluracil levels and TMD (β = 0.069, *P* = 5.11 × 10^–5^). Our findings revealed the causal relationships between immunophenotypes and plasma metabolites on TMD from a genetic perspective, potentially aiding in TMD prevention.

## Introduction

Temporomandibular disorders (TMD) are a subset of craniofacial pain problems impacting the temporomandibular joint (TMJ), masticatory muscles, and other musculoskeletal tissues in the head and neck. According to the National Institute of Dental and Craniofacial Research, the prevalence of TMD ranges from 5 to 12%. The most common symptoms of TMD are pain, asymmetric or limited mandibular motion, and TMJ sounds^1^. Prioritizing conservative treatment modalities will help prevent irreversible interventions as much as possible^2^. The etiology of TMD is still unclear, thus continued exploration is of significant importance.

Over the past few years, the role of the immune system and metabolites in TMD has garnered increasing attention. Immune cells constitute a crucial component of the immune system, with higher levels of immune-inflammatory indices correlating with reduced success rates following temporomandibular joint arthrocentesis^3^. Research suggests that patients with autoimmune diseases face an elevated risk of temporomandibular joint disorder after orthognathic surgery^4^, while the incidence of allergies is significantly higher in patients with temporomandibular joint osteoarthritis^5^. Additionally, a study has reported the involvement of the Th1/Th17/Th22 immune response in the pathogenesis of temporomandibular joint osteoarthritis^6^. Recent studies also highlight immune processes involved in the development and resolution of myogenous TMD^7^. Metabolomics, as an emerging high-throughput technology, offers a new direction for understanding disease mechanisms^8^. Leveraging the heritability of plasma metabolites, they can be utilized for disease prediction or intervention^9^. Interestingly, a recent case–control study suggests that proteins associated with metabolic processes and immune responses may differentiate between TMD myalgia patients and healthy individuals^10^. These results lead us to assume that plasma metabolites and specific immunophenotypes are causally linked to TMD risk. However, previous observational studies have yet to conclusively establish causality between immune features or metabolites and TMD.

Mendelian randomization (MR), utilizing genetic variation to infer causality between exposure and outcome, offers a robust approach to mitigate inherent biases such as reverse causation and confounding in observational studies^11^. Recent advancements in omics-based data and large-scale genome-wide association studies (GWAS) have propelled the advancement of MR studies^12^. In this study, we aim to use comprehensive two-sample MR analysis to explore the potential causal links between immunophenotypes, plasma metabolites, and TMD.

## Materials and methods

### Study design

**Fig. 1:**
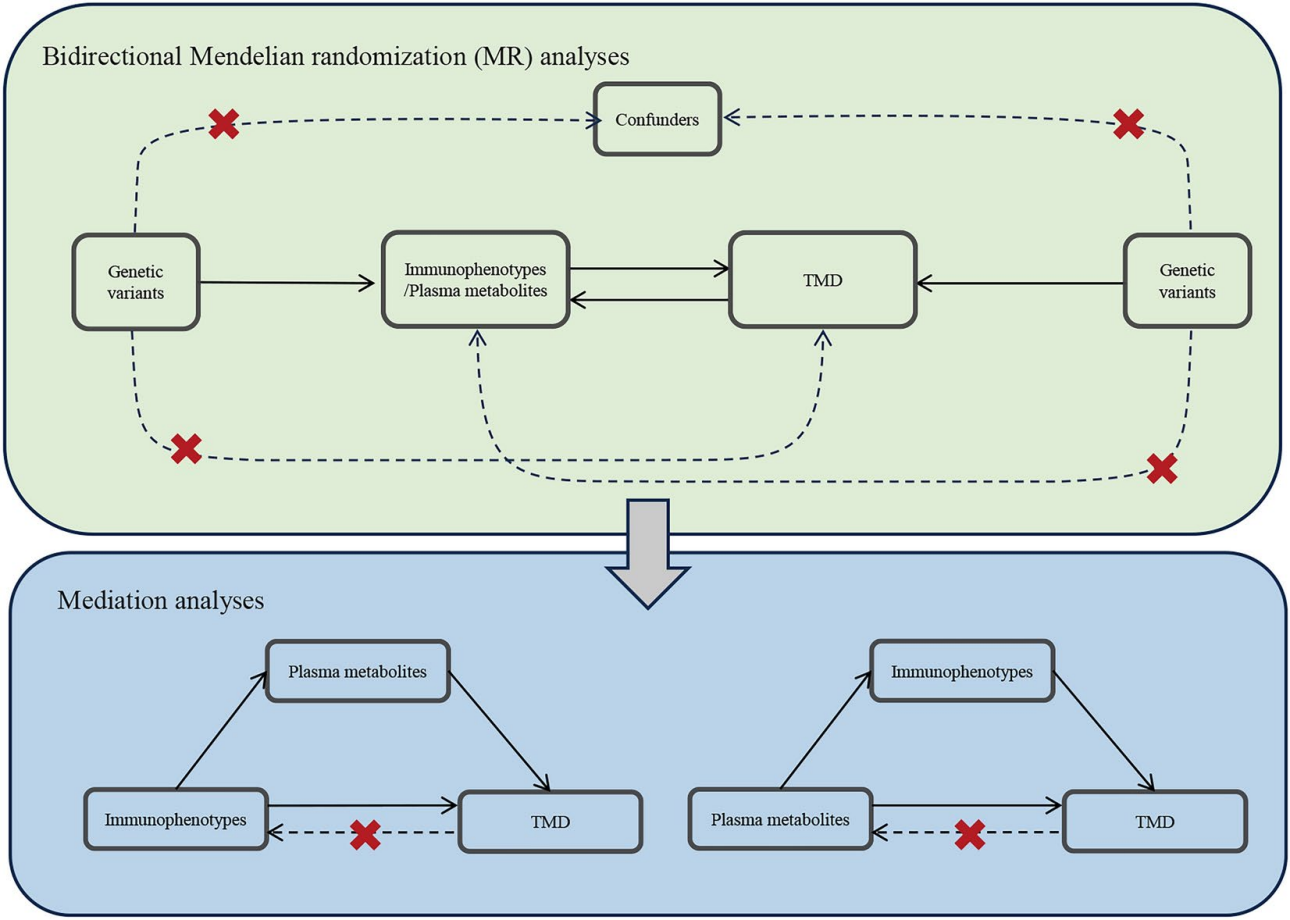
The schematic diagram of the study design. The bidirectional MR analysis identified immunophenotypes and plasma metabolites causally associated with TMD. Mediation analysis further quantified the potential influence of immunophenotypes and plasma metabolites on TMD.

This study is based on a two-sample MR design, an observational epidemiological approach leveraging genetic variants as instrumental variables (IVs) to infer causal relationships between exposures (immunophenotypes and plasma metabolites) and outcomes (TMD). Following the latest MR analysis guidelines outlined in the STROBE-MR checklist^13^, the research is conducted under three fundamental assumptions. Specifically, the genetic variation used as IVs should: (1) exhibit a strong correlation with the exposure factor, (2) be independent of confounding factors, and (3) exert an effect on the outcome only through the exposure factor. The study design is visually represented in Fig. 1.

### Data sources

We selected the largest GWAS conducted to date on immunophenotypes and plasma metabolites^14,15^. The summary statistics for immunophenotypes were obtained from the GWAS Catalog (accession numbers: GCST0001391 to GCST0002121). A total of 731 immune traits were assessed in a cohort of 3757 European individuals from Sardinia. These traits encompassed 118 measurements of absolute cell counts (AC), 389 median fluorescence intensities (MFI) indicative of surface antigen levels, 32 morphological parameters (MP), and 192 relative cell counts (RC). Plasma metabolite data were acquired from a recent GWAS conducted as part of the Canadian Longitudinal Study on Aging. This study involved 8299 European participants and analyzed approximately 15.4 million single-nucleotide polymorphism (SNP) data points, investigating a total of 1091 metabolic features across nine classes. The GWAS data for TMD were sourced from the latest R9 version release by the FinnGen Consortium (https://www.finngen.fi/en), which included 5668 cases and 205,355 controls from European individuals^16^. TMD cases were defined by the ICD-10 code K07.6 (https://risteys.finngen.fi/endpoints/TEMPOROMANDIB), encompassing diagnoses such as “Costen syndrome,” “Temporomandibular joint derangement,” “Snapping jaw,” and “Temporomandibular joint-pain-dysfunction syndrome.” Cases involving “dislocation” and “strain” were excluded. Supplementary Table 1 provides additional detailed information on the specific data sources for this study. All data used in this study were obtained from public publications; therefore, ethical approval or patient consent was not required for analysis.

### Selection of instrumental variables

Based on recent reports in immunology-related studies, considering the rarity of SNPs with a *p*-value less than 5 × 10^–8^, we have set a relatively lenient significance threshold at 1 × 10^–5^. For both plasma metabolites and TMD, we employed the significance level (*p* < 5 × 10^–6^) to select genetic IVs for MR analysis. The criteria for removing linkage disequilibrium involved the utilization of the 1000 Genomes European Sample Data^17^. Furthermore, a clumping threshold of R^2^ < 0.1 was established, and the region width was defined as 500 kb. Weak instruments were eliminated by excluding those with an F-statistic below 10^18^. To ensure result stability, we additionally restricted the inclusion to exposures with a minimum of three SNPs as IVs. Meanwhile, we utilized the PhenoScanner V2 website (http://www.phenoscanner.medschl.cam.ac.uk) to assess secondary phenotypes associated with each SNP and exclude those linked to potential confounders such as pain or psychosocial conditions^19^.

### Main Mendelian randomization analyses

Initially, bidirectional MR analyses were conducted to examine the causal relationships between immunophenotypes and TMD, as well as between plasma metabolites and TMD. The inverse-weighted variance (IVW) method served as the primary approach for assessing causal associations^20^. To address potential type I errors stemming from multiple hypothesis testing, we applied the Benjamini–Hochberg method for False Discovery Rate (FDR) adjustment of *P*-values. Additionally, we employed the MR-Egger^21^, weighted median^22^, weighted mode, and simple mode methods to assess the reliability and stability of results. A positive outcome was defined as significant findings with the primary method and consistent directional effects across all five methods.

### Mediation analysis

To explore potential mediated causal relationships, we further conducted two-step MR analyses^23^. In addition to assessing the total effect of exposure on TMD (β1) obtained from the univariable MR analysis, we evaluated the causal effect of exposure on mediator (β2) and the mediator on TMD (β3). The mediation effect was calculated as the product of β2 and β3.

### Sensitivity analysis

Sensitivity analyses were conducted to evaluate the robustness of causal estimates. Heterogeneity was assessed using Cochran’s Q test. MR-Egger was utilized to estimate horizontal pleiotropy based on its intercept^24^. Additionally, MR-PRESSO was employed to evaluate overall horizontal pleiotropy and identify and remove outliers based on their impact on heterogeneity^25^. A leave-one-out analysis was performed to determine whether individual SNPs significantly influenced the MR estimates.

MR analyses were performed using the “TwoSampleMR” (version 0.5.7), “MendelianRandomization” (version 0.9.0), and MR-PRESSO (version 1.0) packages in R (version 4.2.2)^25,26^.

## Results

### Genetic instruments for exposures

After excluding 309 groups of metabolite ratios data, 1091 blood metabolites data and 731 immunophenotypes were included in the statistical analysis. All IVs exhibited F-statistics exceeding 10, ensuring the strength of the instruments and minimizing biases due to weak instruments. Detailed information on all IVs included in this study is available in Supplementary Tables 2–5.

### Causal estimates between immunophenotypes and TMD

**Fig. 2:**
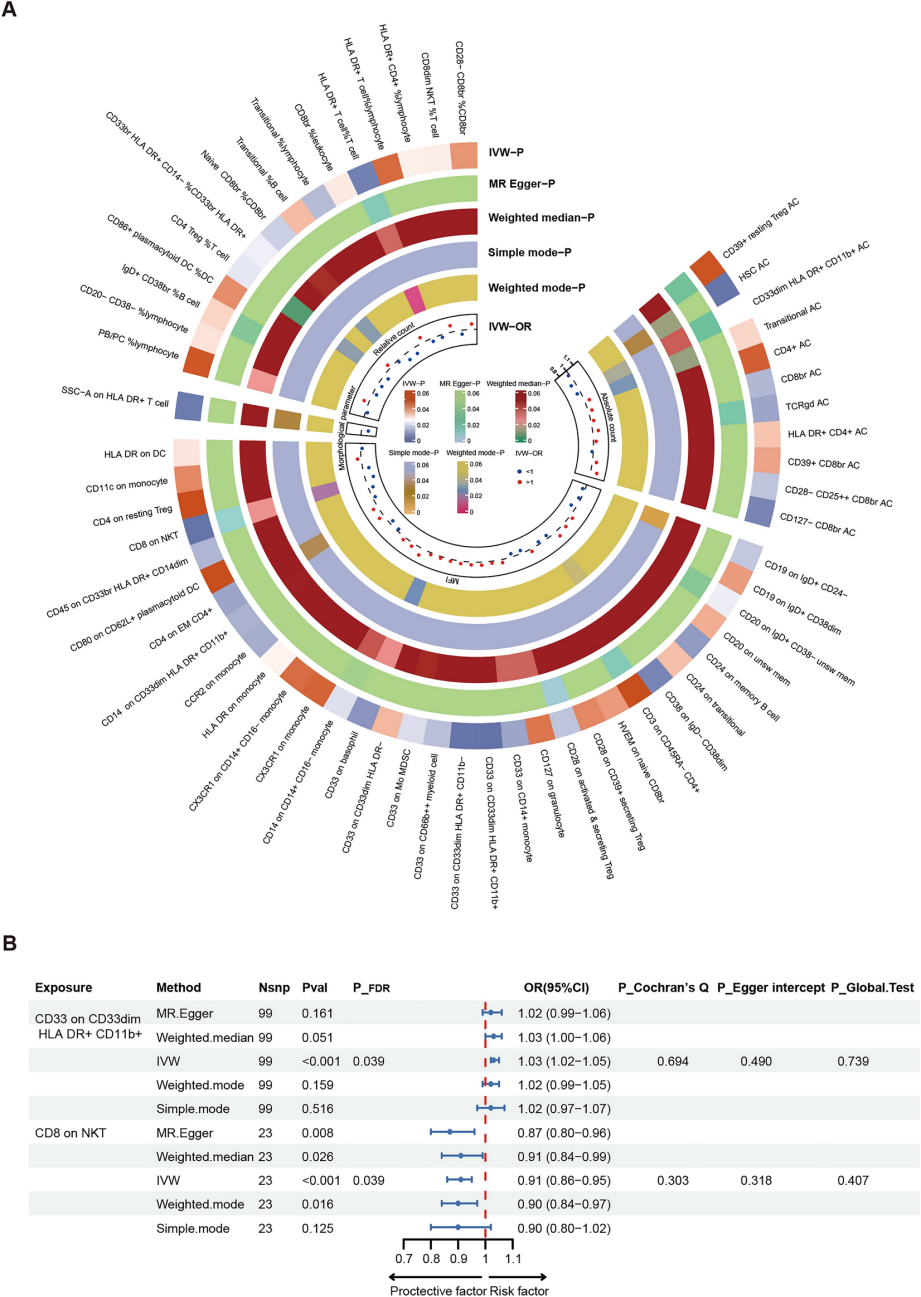
Mendelian randomization analyses show causal effects between immunophenotypes and TMD. (**A**) Circular Heatmap of immunophenotypes associated with TMD at nominal significance. (**B**) Forest plot of immunophenotypes significantly associated with TMD after multiple test adjustment.

In the primary Mendelian randomization analysis, we investigated the causal relationships between genetically predicted 731 immunophenotypes and TMD (Supplementary Table 6). Among them, 59 immunophenotypes exhibited a nominally significant causal association with TMD (IVW,* P* < 0.05). Specifically, these comprised 11 correlations with absolute count, 32 correlations with median fluorescence intensity (MFI), 1 correlation with morphologic parameters, and 15 correlations with relative count (Fig. 2A).

Following adjustment for multiple testing using the FDR method, we identified two immunophenotypes with suggestive significance: CD33 on CD33dim HLA DR + CD11b + (OR_IVW_ = 1.03 [1.02–1.05], *P* = 5.95 × 10^–5^, *P*_FDR_ = 0.039) and CD8 on Natural Killer T (OR_IVW_ = 0.91 [0.86–0.95], *P* = 1.06 × 10^–4^, *P*_FDR_ = 0.039). The results obtained from four additional Mendelian randomization methods, namely MR-Egger, weighted median, weighted mode, and simple mode, consistently supported the trend observed with the IVW method. In terms of sensitivity analysis, no statistically significant evidence of horizontal pleiotropy or heterogeneity was found after excluding outliers (*P*_Egger intercept_ > 0.05, *P*_PRESSSO global test_ > 0.05, and *P*_Cochran’s Q_ > 0.05) (Fig. 2B).

After multiple corrections, the results of the reverse MR analysis also indicate no evidence of a reverse causal relationship between TMD and immunophenotypes (Supplementary Table 7).

### Causal estimates between plasma metabolites and TMD

**Fig. 3:**
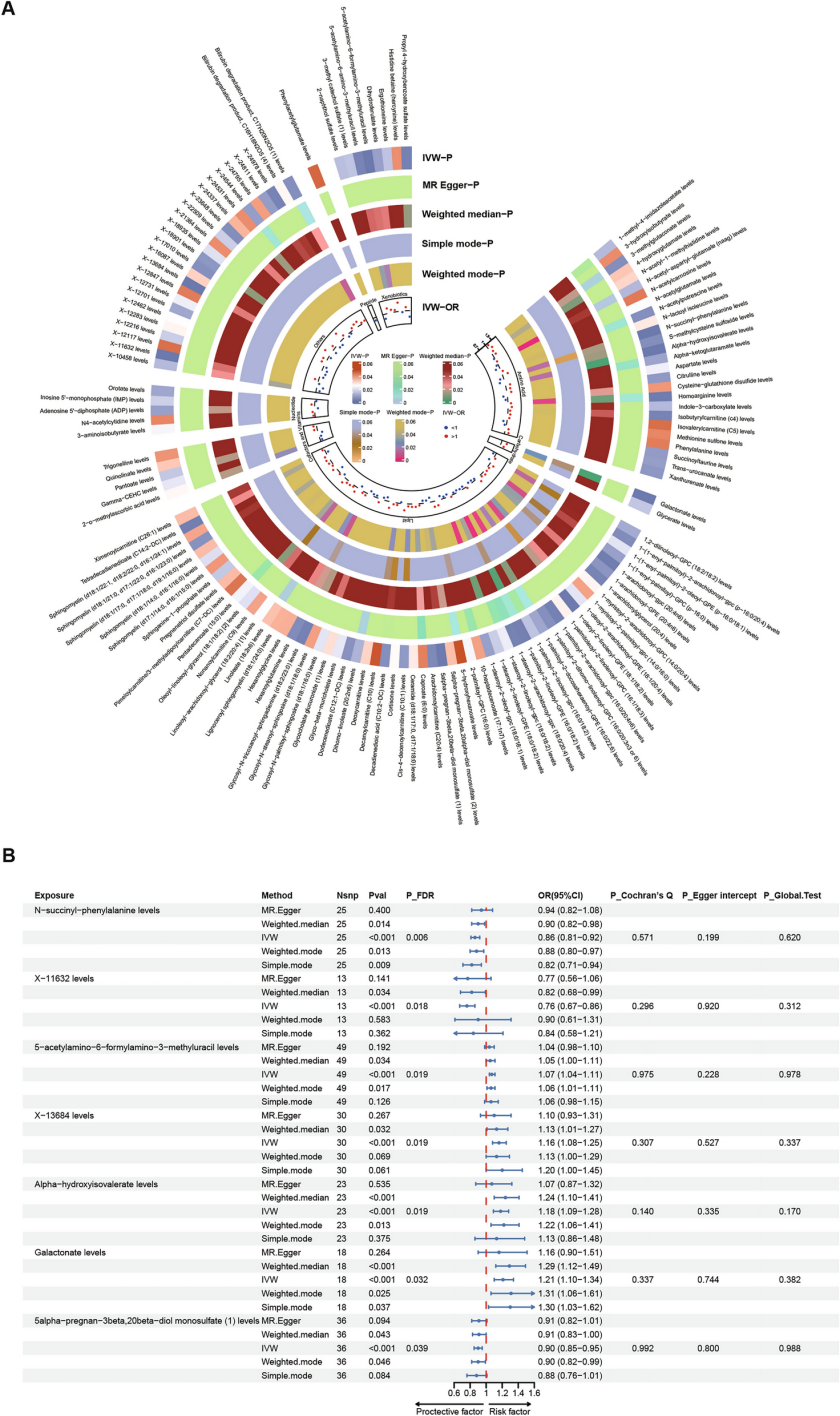
Mendelian randomization analyses show causal effects between plasma metabolites and TMD. (**A**) Circular Heatmap of plasma metabolites associated with TMD at nominal significance. (**B**) Forest plot of plasma metabolites significantly associated with TMD after multiple test adjustment.

Following quality control, we evaluated bidirectional causal relationships between 1091 plasma metabolites and TMD (Supplementary Table 8). A total of 131 metabolites exhibited significant causal relationships with TMD. These included 26 Amino Acids, 5 Cofactors and Vitamins, 2 Carbohydrates, 5 Nucleotides, 59 Lipids, 1 Peptide, 8 Xenobiotics, and 25 Other metabolites (Fig. 3 A).

Among these, seven plasma metabolites withstood multiple testing corrections. The following four metabolites were associated with an increased risk of TMD: 5-acetylamino-6-formylamino-3-methyluracil (OR_IVW_ = 1.07 [1.04–1.11], *P* = 5.11 × 10^–5^, *P*_FDR_ = 0.019), X-13684 (OR_IVW_ = 1.16 [1.08–1.25], *P *= 7.29 × 10^–5^, *P*_FDR_ = 0.019), Alpha-hydroxyisovalerate (OR_IVW_ = 1.18 [1.09–1.28], *P* = 8.89 × 10^–5^, *P*_FDR_ = 0.019), and Galactonate (OR_IVW_ = 1.21 [1.10–1.34], *P* = 1.76 × 10^–4^, *P*_FDR_ = 0.032). And three plasma metabolites were demonstrated a significant effect prevent the progression of TMD: N-succinyl-phenylalanine (OR_IVW_ = 0.86 [0.81–0.92], *P* = 5.92 × 10 –6 , *P*_FDR_ = 0.0065), X-11632 (OR_IVW_ = 0.76 [0.67–0.86], *P* = 3.24 × 10^–5^, *P*_FDR_ = 0.018), and 5alpha-pregnan-3beta,20beta-diol monosulfate (1) (OR_IVW_ = 0.90 [0.85–0.95], *P* = 2.53 × 10^–4^, *P*_FDR_ = 0.039). Sensitivity analysis validated the robustness of the MR results, revealing significant associations for seven plasma metabolites. Moreover, no statistically significant evidence of horizontal pleiotropy or heterogeneity was observed in sensitivity analysis, as indicated by *P*-values exceeding 0.05 for the Egger intercept, MR-PRESSO global test, and Cochran’s Q test (Fig. 3B).

Ultimately, reverse MR was conducted for TMD and plasma metabolites. Following multiple corrections and sensitivity analysis, no significant and stable results were discerned (Supplementary Table 9).

### Mediation analyses link “immunophenotypes, plasma metabolites and TMD”

**Fig. 4:**
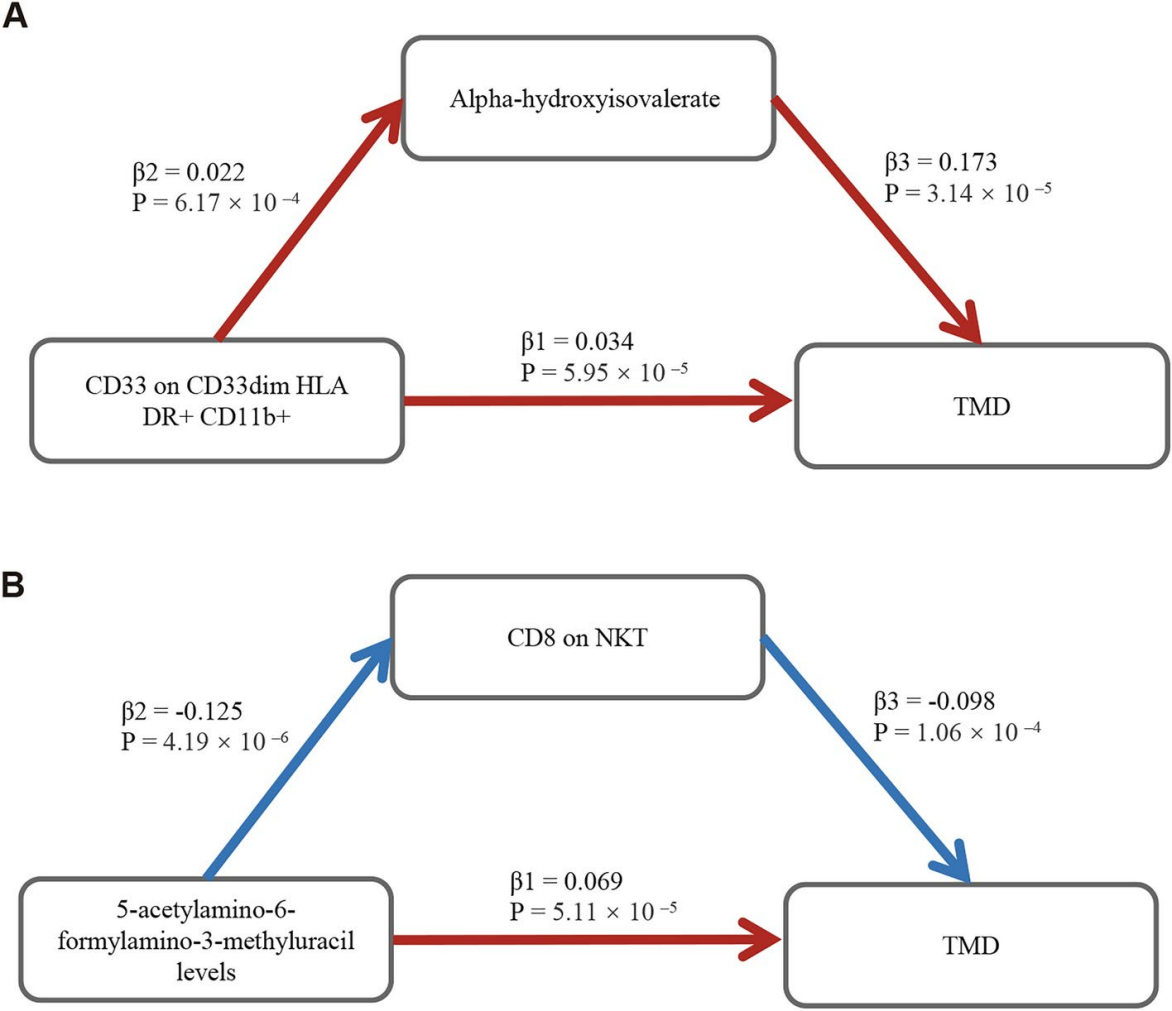
Potential mediated causal relationships of immunophenotypes and plasma metabolites on TMD. (**A**) Mediation effect of Alpha-hydroxyisovalerate in the causal association between CD33 on CD33dim HLA DR + CD11b + and TMD. (**B**) Mediation effect of CD8 on NKT in the causal association between 5-acetylamino-6-formylamino-3-methyluracil levels and TMD.

Having identified two immune cell phenotypes and seven plasma metabolites with potential causal relationships with TMD, we subsequently performed a two-step MR to investigate the potential mediated causal relationships. Following corrections for multiple testing, we identified two potential mediation relationships. The positive causal effect of CD33 on CD33dim HLA DR + CD11b + on TMD was mediated by Alpha-hydroxyisovalerate levels (indirect effect = 11.3%) (Fig. 4 A). Additionally, the positive causal effect of 5-acetylamino-6-formylamino-3-methyluracil levels on TMD is mediated by CD8 on NKT (indirect effect = 17.7%) (Fig. 4 B). Besides, the reverse MR analyses led to the following conclusions: (i) Alpha-hydroxyisovalerate levels do not predict CD33 expression on CD33dim HLA DR + CD11b + cells. (ii) TMD does not predict alpha-hydroxyisovalerate levels. (iii) CD8 on NKT is not predictive of 5-acetylamino-6-formylamino-3-methyluracil levels. (iv) TMD is not predictive of CD8 on NKT.

## Discussion

This study employed large-scale GWAS summary data and applied a comprehensive two-sample MR approach to examine the causal relationship between immunophenotypes and plasma metabolite on TMD. After rigorous multiple corrections and sensitivity analyses, we identified two immunophenotypes and seven plasma metabolites that exhibit significant causal relationships with TMD. Additionally, mediation Mendelian randomization analysis revealed potential mediated causal relationships among these factors. Our findings offer new insights into the complex mechanisms underlying TMD pathogenesis, particularly highlighting the involvement of immune-metabolic interactions.

The etiology of TMD is complex, and our study underscores the intricate relationship between immune cells and TMD risk, consistent with prior research^1,27,28^. We identified a positive causal relationship between CD33 on CD33dim HLA DR + CD11b + and TMD, alongside a negative causal relationship between CD8 on Natural Killer T cells and TMD. CD33, also known as Sialic-acid-binding immunoglobulin-like lectin (Siglec), plays a critical role in mediating cell–cell interactions and maintaining immune cells in a resting state^29–31^. The connection between CD33 and the De Ley-Dourdoroff pathway is particularly noteworthy, as CD33 is involved in the utilization of galactose for sialic acid synthesis, thereby linking it to Galactonate metabolism. This association suggests that alterations in post-transcriptional protein glycosylation, specifically through sialic acid pathways, may be a key mechanism contributing to pain development in TMD. This mechanism could have broader implications, affecting not only immune cells but also muscle, bone, and connective tissue, all of which are integral to TMD pathology. Natural Killer T (NKT) cells play a crucial role in immune regulation, contributing to tissue homeostasis and infection prevention^32^. Numerous studies highlight their pivotal role in immunity against tumors, bacteria, viruses, and suppression of cell-mediated autoimmunity^33–35^. Additionally, NKT cells offer protection against joint disorders, including rheumatoid arthritis, and mitigate chronic joint inflammation post-Borrelia burgdorferi infection^36,37^. Notably, as inflammation subsides in myogenous TMD, there is a significant increase in gene expression associated with NK, NKT, and T cell activation and accumulation^7^.

In recent years, emerging evidence points to the significant involvement of metabolites in TMD^38–42^. Our study identified several metabolites with significant causal relationships to TMD, both positive and negative. Notably, we found that Alpha-hydroxyisovalerate, a metabolite with origins in the degradation of branched-chain amino acids and fatty acid oxidation, is positively associated with TMD risk. The mediation analysis further suggests that Alpha-hydroxyisovalerate levels may mediate the causal effect of CD33 on TMD. This finding reinforces the hypothesis that immune-metabolic dysregulation, particularly involving sialic acid metabolism, plays a critical role in the development of TMD. Galactonate, another key metabolite identified in our study, is involved in the De Ley-Dourdoroff pathway, a less commonly explored metabolic pathway in human physiology. Its association with TMD suggests increased activity or potential inhibition points within this pathway, possibly linked to altered glycosylation processes. Given the reduced levels of D-galactose observed in TMD patients^43^, it is plausible that Galactonate formation from D-galactose may contribute to increased TMD risk by interfering with normal glycosylation processes essential for tissue integrity and immune function. Furthermore, our study highlights the protective roles of certain metabolites, such as N-succinyl-phenylalanine, which is involved in phenylalanine metabolism and associated with systemic effects like blood pressure regulation^44,45^. These findings suggest that metabolic pathways influencing systemic physiology may also impact TMD risk, further underscoring the complex interplay between metabolism and immune regulation in TMD.

This is the first comprehensive MR study to evaluate the relationships between immune features, plasma metabolites, and TMD. The utilization of the MR design with the largest publicly available GWAS datasets is a significant strength of our work. Rigorous multiple corrections and sensitivity analyses ensure the reliability of our results. However, it is imperative to acknowledge certain limitations. Firstly, our study adopts a more liberal significance threshold for selecting genetic IVs to facilitate comprehensive results and sensitivity analysis. Secondly, the inclusion of only individuals of European descent necessitates caution in extrapolating results to other ethnic or racial groups. Lastly, the lack of experimental validation of the identified causal relationships highlights the need for future mechanistic studies to confirm these findings.

Future research should focus on validating these causal relationships through experimental and longitudinal studies, particularly in diverse populations. Additionally, further exploration of the molecular mechanisms underlying immune-metabolic interactions, specifically involving sialic acid pathways and protein glycosylation, could provide deeper insights into TMD pathophysiology and reveal novel therapeutic targets.

## Conclusion

In conclusion, our comprehensive bidirectional MR analysis has unveiled potential causal relationships between immunophenotypes, plasma metabolites, and TMD. These findings not only advance our understanding of the underlying mechanisms of TMD but also hold promise for informing novel approaches to prediction and treatment strategies for this condition.

## Supplementary Information

Supplementary Information.

## Data Availability

The datasets used during the current study are available from public databases and previous studies. Further information is available from the corresponding author on reasonable request.
